# A case of early neonate bovine tuberculosis in Ethiopia

**DOI:** 10.1002/ccr3.3563

**Published:** 2020-11-29

**Authors:** Getnet Abie Mekonnen, Balako Gumi, Stefan Berg, Andrew J. K. Conlan, Gobena Ameni, James L. N. Wood

**Affiliations:** ^1^ National Animal Health Diagnostic and Investigation Center Sebeta Ethiopia; ^2^ Aklilu Lemma Institute of Pathobiology Addis Ababa University Addis Ababa Ethiopia; ^3^ Animal and Plant Health Agency Weybridge UK; ^4^ Disease Dynamics Unit Department of Veterinary Medicine University of Cambridge Cambridge UK; ^5^ Department of Veterinary Medicine College of Food and Agriculture United Arab Emirates University Al Ain United Arab Emirates

**Keywords:** bovine neonate, ethiopia, *Mycobacterium bovis*, tuberculous lesion

## Abstract

This report illustrates that calves may be infected with bovine tuberculosis at early age under natural conditions and progression can be rapid. Thus, testing of calves needs to be considered in any control program to reduce the risk of transmission.

## Graphical abstract

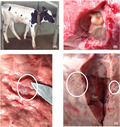

## 1 INTRODUCTION

Bovine tuberculosis is a chronic disease rarely observed in an early age. Our observation of tubercle lesions in the lungs of a three‐week‐old calf and confirmation of *Mycobacterium bovis* strains illustrate that the progression of tuberculosis in neonates can be rapid under natural conditions, contributing to transmission within herds.

Bovine tuberculosis (bTB) is a chronic disease, caused primarily by *M bovis*, affecting cattle, other domesticated animals, certain wildlife species, nonhuman primates, and humans.^1^, ^2^, ^3^ Cattle can be infected at any age, but tubercle lesion development and subsequent clinical signs may appear at a later stage. Calves can be born with tuberculosis congenitally when infected with *M bovis* either via the umbilical vein from an infected genital tract of the dam, from the infected placenta, or from infected amniotic fluid.^4^ After birth, neonates can also be infected even at an early stages following delivery, mainly by inhaling infected droplets and/ or by ingesting raw milk or colostrum from infected cows. Other routes of infection such as cutaneous, though not common, are still important in situations where the prevalence of bTB is high.^5^ As the course of the disease is slow compared with many other infectious diseases, infected animals can spread bTB to other herd mates and newborns before it begins to manifest clinical signs. The transmission rate may be faster at late stages of infection as the number of organisms being shed is larger in this stage.^1^

The observation of characteristic tubercle lesions in the lungs of a three‐week‐old male calf led us to raise questions like “how the calf could get the infection?” and “how fast is the disease progression under natural infection?”. In order to get answers to these questions, further investigation was made and this case report was compiled from the pathological and molecular investigations conducted for the diagnosis of the case.

## 2 CASE HISTORY

A dairy farm located in northern Ethiopia and with a herd size of 100 cattle (86 crossbreeds of Holstein‐Frisian and Zebu breeds and 14 Zebu breeds) was screened for bTB by the single intradermal cervical comparative tuberculin (SICCT) test. As it was learned from its history, the farm was newly established just a year earlier through the purchase of cows and heifers from dairy farms and markets in Mekelle City and nearby towns, including Adigrat and Humera. The SICCT testing was conducted on the herd in 2016 by Mekonnen et al,^6^ and a prevalence of 12% (95% CI: 7%‐19.8%) was recorded. One of the reactors was a three‐week‐old calf (ID 8A0597), and its skin induration was 8 mm according to the calculation of the SICCT test result. In the same week, the SICCT test was conducted, all positive reactors (including the calf) were culled following disclosure of the test results to make the herd clear from bTB. It was observed from the husbandry of the farm that all calves in the farm were sharing the same housing with the adults. Moreover, the calves were fed on pooled milk obtained from milking cows in the herd including one SICCT reactor cow and looked after by the same individuals who were looking after the adult cattle.

The three‐week‐old calf positive for the SICCT test drew our attention. The calf had moderate body condition, but it was depressed and characterized by hair loss on various parts of its body. The dam of the calf (ID 8A0417) was a 10 years old cross of Holstein‐Frisian and Zebu breeds. The body condition of this cow was rated as “Good,” and it was negative for bTB by the SICCT test according to the interpretation recommended by OIE.^7^ In addition, nasal discharge was collected from the dam using a cotton swab designed for such purpose, and the PBS wash of the swab was centrifuged at ‐3100 g for 15 min. Genomic DNA was extracted from the pellet using a Qiagen DNA extraction kit as per the manufacturer's instruction,^8^ and the eluent tested by an RD4‐based PCR assay for specific identification of *M bovis*. The primers defined by Brosch et al^9^ were used in the PCR assay procedure described by Berg et al^10^

## 3 INVESTIGATION

**Figure 1:**
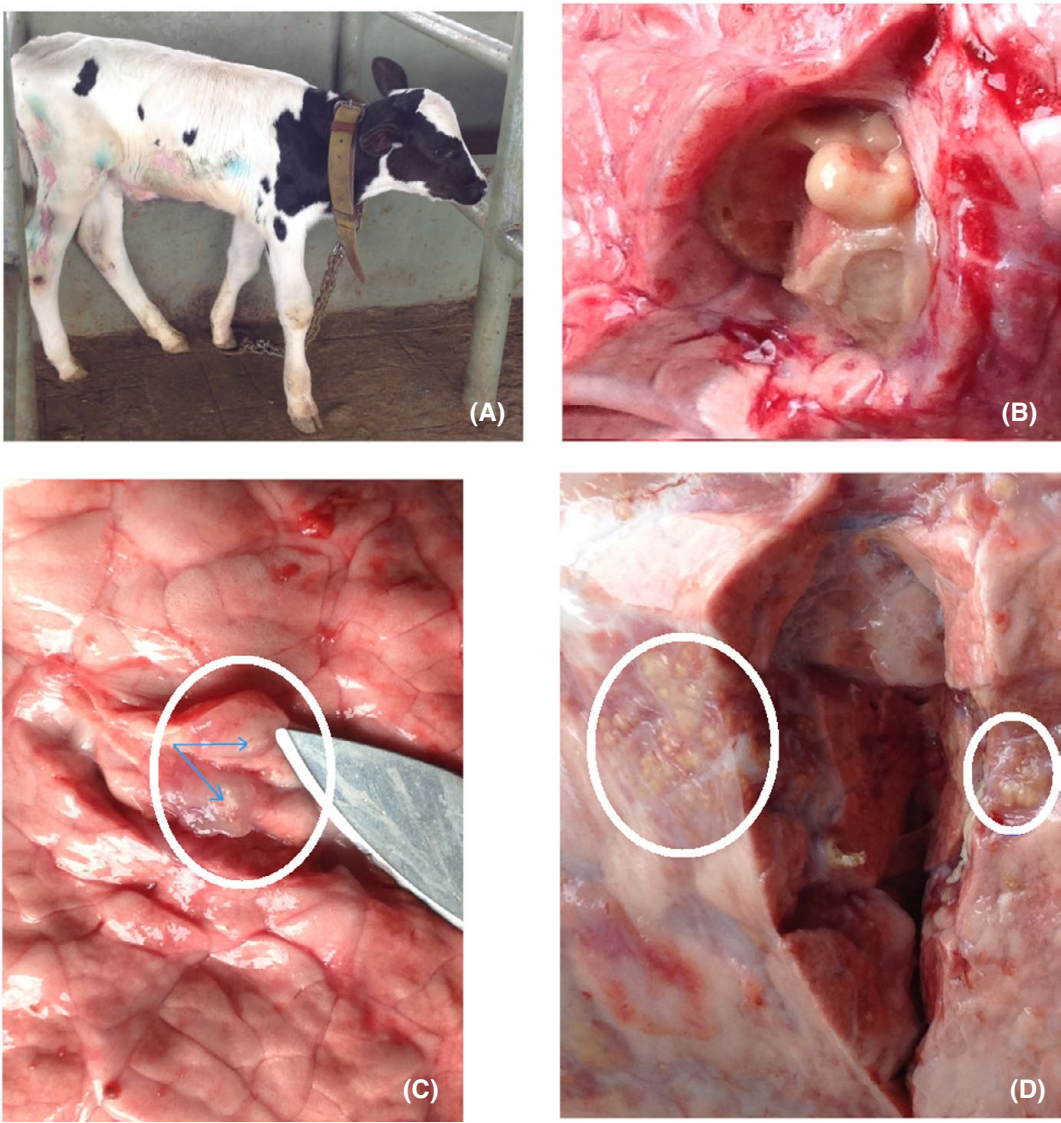
Gross tubercle lesions in the lung lobes of a three‐week‐old SICCT reactor calf born from a 10‐year‐old cow (A). Loosely encapsulated structure [diaphragmatic lobe] (B); lung tissue [apical lobe]—granuloma‐like lesions (C); lung tissue [apical lobe] multiple granuloma, circumscribed in circles (D)

During postmortem inspection of the calf, multiple granulomas were observed in the apical lobe of the lungs (Figure 1). A single ~20 mm diameter of a loosely encapsulated granuloma‐like structure was also observed in the diaphragmatic lobe. Lymph nodes and other vital organs appear normal.

**Figure 2:**
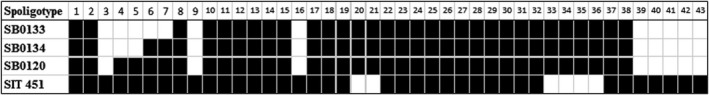
Spoligotype patterns of two Mycobacterium bovis strains isolated from lung tissues of a three‐week‐old reactor calf to the SICCT test. The spoligotype patterns are shown here as 43 spacers in the Direct Repeat region11, either being present (Black) or absent (White). Positive controls: SB0120 (Mycobacterium bovis) and SIT451 (Mycobacterium tuberculosis H37Rv)

The culturing result (Löwenstein‐Jensen medium slant) from lung tubercles of the calf showed two strains of typical *M bovis*. Heat‐killed colonies were tested by RD4‐based PCR and confirmed that both strains were *M bovis*. Spoligotyping^11^ then classified these two strains into different spoligotype patterns, SB0134 and SB0133 (Figure 2). Genomic DNA extracted from the dam's nasal discharge was positive for *M bovis* by the RD4‐based PCR assay; however, spoligotyping was not sensitive enough to confirm the pattern, and the DNA concentration was too low for MIRU‐VNTR typing or whole genome sequencing. Attempt to culture the agent from the nasal swab sample of the dam was not successful.

## 4 DISCUSSION

*Mycobacterium bovis* strains identified in the present case, classified as spoligotypes SB0134 and SB0133, suggesting mixed infection. Although mixed infection has not been frequently described in Ethiopia, it is more likely to happen in herds which have established their stocks from different farms, as was the case for the present herd. Hence, the mixed infection observed in this calf could be due to transmission from more than one reactor, possibly recruited from different herds without prior testing for bTB.

The time‐frame for development of tubercle lesions in the investigated calf is comparable with artificially infected calves via respiratory route under experimental conditions.^12^ However, under such conditions; the extent of pathology could vary depending on the infective dose and factors specific to the animals and husbandry systems.

Evidence has shown that the route of transmission of *M bovis* could be inferred from lesions distribution.^13^ In the present calf, tubercle lesions were localized in the lungs and no tubercle lesions were observed in other tissues and organs, which could suggest inhalation of infectious droplets as a possible route of infection while ingestion of infected colostrum or milk might seem less likely. Other possibilities of in utero transmission through hematogenous route and/or aspiration of *M bovis* contaminated amniotic fluid^14^, ^15^ could not be justified as there was no lesion suggestive of bTB in other vital organs, such as liver. Tuberculosis transmitted hematogenously is typically characterized by the presence of hepatic tubercles or more disseminated form of the disease,^16^ while through aspiration of infected amniotic fluid, the primary tubercle could be in the lungs and/or the gut.

The molecular test result implies that the dam was infected with *M bovis* even though it was not a reactor to the SICCT test (the increase at the PPD‐B injection site was 3.2 mm while at the PPD‐A site it was 2.9 mm). This could potentially be due to a development of an anergic status, in which the dam would either had been in a late disease stage or immunosuppressed attributable to the hormonal effects during pre‐ and post‐parturition. An animal with a chronic stage of the disease is known to be highly infectious and would likely serve as a good source of infection to a neonate by transmission in several possible ways.^17^, ^18^ Whether or not this was the case for the observed dam, it is still likely that her calf got infected in its first week of life, while suckling the colostrum and/or inhalating the aerosol produced by her. Attempt to generate strong evidence to establish transmission from the dam has not been successful due to failure to culture *M bovis* from the nasal swab sample; resampling of the dam was not possible as the animal was later culled without the knowledge of the authors. Despite the proximity and connection between the dam and its calf, the possibility of transmission from other neighboring infected animals could not be ruled out as all reactors in this farm shared the same housing and husbandry system; this could also explain why two strains of different spoligotype patterns were isolated from its lungs.

In conclusion, although this is a single case, it illustrates that the progression of bTB in calves can be rapid under natural conditions, contributing to transmission within herds. This has important implications in the appropriate structure of models used to estimate the rate of transmission within herds.^19^ Calf can respond to the tuberculin test at a very early age and thus should be considered in the routine bTB screening to reduce transmission within herds.

## CONFLICT OF INTEREST

The authors declare that they have no competing interests.

## AUTHORS’ CONTRIBUTION

GAM performed the investigation, analyzed the data, and wrote the report. BG, SB, AJKC, GA, and JLNW contributed to the study conception and design and reviewed the report.

## ETHICAL APPROVAL

This study was under the framework of a research protocol approved by the National Research Ethics Review Committee ‐ NRERC No. 3.10/800/07.

## Data Availability

All data generated and/or analyzed during this study are not publicly available but are available from the corresponding author on reasonable request.
